# Supplementary material for: A computational workflow for the expansion of heterologous biosynthetic pathways to natural product derivatives
Source: Nat Commun. 2021 Mar 19;12:1760. doi: 10.1038/s41467-021-22022-5 (PMC7979880; doi:10.1038/s41467-021-22022-5)
Supplement: Supplementary file 1 — Supplementary Information [file 41467_2021_22022_MOESM1_ESM.pdf]

A computational workflow for the expansion of heterologous biosynthetic pathways to natural product derivatives

Hafner and Payne *et al.*

Supplementary Table 1. Overview on network statistics.

	Reactions	Compounds
Total entries in raw BNICE.ch network	17,597	4,838
Biological / bioactive entries in raw BNICE.ch network	244	720
Total entries in benzylisoquinoline alkaloid network	7,527	1,518
Biological / bioactive entries in benzylisoquinoline alkaloid network	49	99
Potential targets		1,501
Potential targets with annotation (min 1 citation/patent)		545

Supplementary Table 2. Presence of known biosynthetic BIA pathways in the generated reaction network.

Compound name	Source database	In net-work	Precursor[a]	Shortest pathway[b]	Shortest pathway in source[c]	Pathways visualized online	Comment
Allocryptopine	KEGG	yes	(*S*)-cis-*N*-methylcanadine	1	1	yes	
Berberine	MetaCyc	yes	(*S*)-canadine	1	1	yes	
Dehydroscoulerine	MetaCyc	yes	(*S*)-scoulerine	1	1	no	
(*S*)-nororientaline	KEGG	yes	3'-Hydroxy-*N*-methyl-(*S*)-coclaurine	2	2	no	
Corydaline	KEGG	yes	Tetrahydro-columbamine	2	3	yes	Shortcut found via tetrahydrapalmatine or isocorybulbine.
Magnoflorine	KEGG, MetaCyc	yes	(*S*)-reticuline	2	2	yes	
Palmatine	MetaCyc	yes	Tetrahydro-columbamine	2	2	yes	
Salutaridinol	KEGG	yes	(*S*)-reticuline	2	4	yes	2 steps from (*R*)-reticuline, or 4 steps from (*S*)-reticuline.
Coptisine	MetaCyc	yes	(*S*)-scoulerine	3	3	yes	
Epiberberine	MetaCyc	yes	(*S*)-scoulerine	3	3	yes	
Protopine	KEGG	yes	(*S*)-cis-*N*-methylcanadine	3	4	yes	Shortcuts found via allocryptopine, hunnemanine, and others.
Papaverine	KEGG, MetaCyc	yes	(*S*)-reticuline	4	4	yes	
Chelerythrine	MetaCyc	no	(*S*)-cis-*N*-methylcanadine	N/A	4		Chelerythrine is 4 reaction steps away from the precursor. Step 1 and 2 are in the network, but step 3 (a spontaneous reaction) is not.

[a]Closest noscapine pathway intermediate; [b]Length of shortest pathway from precursor to compound; [c]Distance from precursor in source database

Supplementary Table 3. Additional information for 15 candidate targets one reaction step away from the initial noscapine pathway.

Rank	Name	Iteration	Identifier	Number of pathways	Precursor	Best BridgIT score	Predicted enzyme
3	Berberine	1	79	2	(*S*)-canadine	1.00	1.3.3.8
6	Tetrahydropalmatine	1	171	2	Tetrahydrocolumbamine	1.00	2.1.1.89
13	Columbamine	1	83	1	Tetrahydrocolumbamine	0.99	1.3.3.8
15	Salutaridine	1	155	1	(*S*)-reticuline	1.00	1.14.19.67
16	Norlaudanosoline	1	113	3	(*S*)-norcoclaurine	0.99	1.14.14.102
17	Stepholidine	1	150	1	Tetrahydrocolumbamine	0.78	1.14.13.31
18	Allocryptopine	1	100	2	(*S*)-cis-*N*-methylcanadine	0.32	1.14.13.239
24	Laudanine	1	169	3	(*S*)-reticuline	1.00	2.1.1.291
31	Codamine	1	98	2	(*S*)-reticuline	0.79	2.1.1.121
33	Norreticuline	1	89	3	(*S*)-reticuline	0.09	1.5.3.10
37	Corytuberine	1	154	1	(*S*)-reticuline	0.56	1.14.19.67
39	Lambertine	1	75	6	(*S*)-canadine	0.45	1.3.1.29
43	Armepavine	1	167	2	(*S*)-*N*-methylcoclaurine	1.00	2.1.1.291
43	1,2-Dehydroreticuline	1	93	1	(*S*)-reticuline	1.00	1.5.1.27
46	Nandinine	1	139	1	(*S*)-scoulerine	1.00	1.14.19.73

Supplementary Table 4. Top 5 BridgIT predictions for the biosynthesis reactions towards laudanine, armepavine, and nandinine.

BridgIT rank	BridgIT score	Predicted EC number	Corresponding enzyme
Laudanine			
1	1.00	2.1.1.291	CjColOMT
2	0.98	2.1.1.89	Ps7OMT
3	0.77	2.1.1.118	Ps7OMT
4	0.75	2.1.1.128	PsHNC4’OMT
5	0.74	2.1.1.121	Ps6OMT
Armepavine			
1	1.00	2.1.1.291	CjColOMT
2	0.98	2.1.1.89	Ps7OMT
3	0.78	2.1.1.118	Ps7OMT
4	0.77	2.1.1.128	PsHNC4’OMT
5	0.76	2.1.1.116	Ps6OMT
Nandinie			
1	1.00	1.14.21.5	AmCYP719A13
2	1.00	1.14.21.1	EcCYP719A3
3	1.00	1.14.21.12	EcCYP719A3
4	0.97	1.14.21.2	EcCYP719A3
5	0.76	1.14.21.11	ShCYP719A23

Supplementary Table 5. Genes used in this work.

Gene ID	Description	Source organism	Accession ID
AtCafOMT	Caffeoyl-coenzyme A *O*-methyltransferase	*Arabidopsis thaliana*	NM_001342249.1
CjColOMT	Columbamine *O*-methyltransferase	*Coptis japonica*	AB073908.1
LjFlaOMT	2,4’,7’-trihydroxyisoflavanone *O*-methyltransferase	*Lotus japonica*	AK339587.1
SaPurOMT	*O*-demethylpuromycin *O*-methyltransferase	*Streptomyces alboniger*	M74560.2
PsBBE	Berberine bridge enzyme	*Papaver somniferum*	AF025430.1
PsS9OMT	Scoulerine 9-*O*-methyltransferase	*Papaver somniferum*	JN185323.1
Ps6OMT	Norcoclaurine 6-*O*-methyltransferase	*Papaver somniferum*	AY268894.1
PsCNMT	Coclaurine *N*-methyltransferase	*Papaver somniferum*	AY217336.1
Ps4’OMT	6-Methyl-(*S*)-laudanosoline 4’-*O*-methyltransferase	*Papaver somniferum*	MF038041.1
Cj4’OMT	6-Methyl-(*S*)-laudanosoline 4’-*O*-methyltransferase	*Coptis japonica*	D29812.1
Ec4’OMT	6-Methyl-(*S*)-laudanosoline 4’-*O*-methyltransferase	*Eschscholzia californica*	AB745041.1
Tf4’OMT	6-Methyl-(*S*)-laudanosoline 4’-*O*-methyltransferase	*Thalictrum flavum*	AY610510.1
CjN6OMT	Norcoclaurine 6-*O*-methyltransferase	*Coptis japonica*	D29811.1
Ps7OMT	(*R*,*S*)-reticuline 7-*O*-methyltransferase	*Papaver somniferum*	AY268893.1
PsHNC4’OMT	3'-Hydroxy-*N*-methylcoclaurine 4'-*O*-methyltransferase	*Papaver somniferum*	AY217333.1
AmCYP719A13	Stylopine synthase	*Argemone mexicana*	EF451151.1
EcCYP719A3	Stylopine synthase	*Eschscholzia californica*	AB126256.1
NnSCNS	Stylopine/canadine/nandinine synthase	*Nelumbo nucifera*	XM_010268782.1
PsCYP719A21	Canadine synthase	*Papaver somniferum*	JQ659003.1
ShCYP719A23	Pluviatolide synthase	*Sinopodophyllum hexandrum*	KC110997.1

Supplementary Table 6. Yeast strains used in this work.

Strain ID	Description	Genotype
CSY1314	*De novo* (*S*)-tetrahydrocolumbamine (THCB) strain	CSY1171[1]; *trp1*∷ P_{PGK1}-*PsBBE*-T_{PHO5}, P_{TEF1}-*yPsS9OMT*-T_{CYC1}
CSY1315	*De novo* THCB strain with *eCj4'OMT*	CSY1314; *Ps4'OMT* ∷ *eCj4'OMT*‡; Δ *yPs4'OMT**
CSY1316	*De novo* THCB strain with *yCj4'OMT*	CSY1314; *Ps4'OMT* ∷ *yCj4'OMT*; Δ *yPs4'OMT**
CSY1317	*De novo* THCB strain with *eEc4'OMT*	CSY1314; *Ps4'OMT* ∷ *eEc4'OMT*‡; Δ *yPs4'OMT**
CSY1318	*De novo* THCB strain with y*Ec4'OMT*	CSY1314; *Ps4'OMT* ∷ *yEc4'OMT*; Δ *yPs4'OMT**
CSY1319	*De novo* THCB strain with *Tf4'OMT*	CSY1314; *Ps4'OMT* ∷ *Tf4'OMT*; Δ *yPs4'OMT**
CSY1320	*De novo* scoulerine strain	CSY1171[1]; *trp1*∷ P_{PGK1}-*PsBBE*-T_{PHO5}
CSY1322	*De novo N*-methylcoclaurine strain	CSY1171[1]; Δ *PsCYP80B1*; Δ *PsCPR*; Δ *Ps4'OMT**

*CSY1171 contains two different isoforms of the *Ps4'OMT* gene – one that is wild-type, called here *Ps4'OMT*, and one that is codon-optimized for the yeast *S. cerevisiae*, called here *yPs4'OMT*

‡ The prefix "e" here denotes that the gene is codon-optimized for expression in *E. coli*

Supplementary Table 7. LC-MS/MS multiple reaction monitoring (MRM) transitions and parameters used in this work.

Compound	Quantifier MRM transition (m/z^+)	Fragmentor voltage	Collision energy	Reference
(*S*)-tetrahydropalmatine	356.2 → 192	170	29	This work
(*S*)-tetrahydrocolumbamine	342 → 178	135	29	[2]
(*S*)-reticuline	330 → 192	120	19	[2]
(*S*)-scoulerine	328 → 151	135	30	[2]
(*S*)-*N*-methylcoclaurine	300 → 107	100	37	[5]
(*S*)-armepavine	314 → 107	100	35	[6]
(*S*)-laudanine	344 → 137	120	35	[7]
(*S*)-nandinine	326 → 176	135	30	[8]

Compound	Qualifier MRM transition (m/z^+)	Fragmentor voltage	Collision energy	Reference
(*S*)-tetrahydropalmatine	356.2 → 176	170	61	This work
(*S*)-tetrahydrocolumbamine	342 → 163	135	29	[2]
(*S*)-reticuline	330 → 137	120	31	[2]
(*S*)-scoulerine	328 → 178	135	29	[2]
(*S*)-*N*-methylcoclaurine	300 → 175	100	25	[9]
(*S*)-laudanine	344 → 189	120	35	[7]
(*S*)-nandinine	326 → 149	135	30	This work

Supplementary Table 8 Exact *p*-values from Student's two-tailed t-tests in Figures 3 & 4.

Figure	Test	*p*-value
3b	PsS9OMT only vs. +CjColOMT	0.0148
3c	Without CjColOMT vs with CjColOMT for eCj4'OMT	1.92×10^{-4}
3c	Without CjColOMT vs with CjColOMT for yCj4'OMT	3.52×10^{-5}
3c	Without CjColOMT vs with CjColOMT for eEc4'OMT	4.91×10^{-4}
3c	Without CjColOMT vs with CjColOMT for yEc4'OMT	2.46×10^{-3}
3c	Without CjColOMT vs with CjColOMT for Tf4'OMT	2.49×10^{-3}
4b	Empty vector vs. yCjColOMT	2.45×10^{-3}
4b	Empty vector vs. yCjN6OMT	0.0362
4c	Empty vector vs. yCjColOMT	4.35×10^{-3}
4c	Empty vector vs. yPsHNC4'OMT	8.95×10^{-3}
4d	Empty vector vs. yEcCYP719A3	3.86×10^{-3}
4d	Empty vector vs. yPsCYP719A21	0.0467

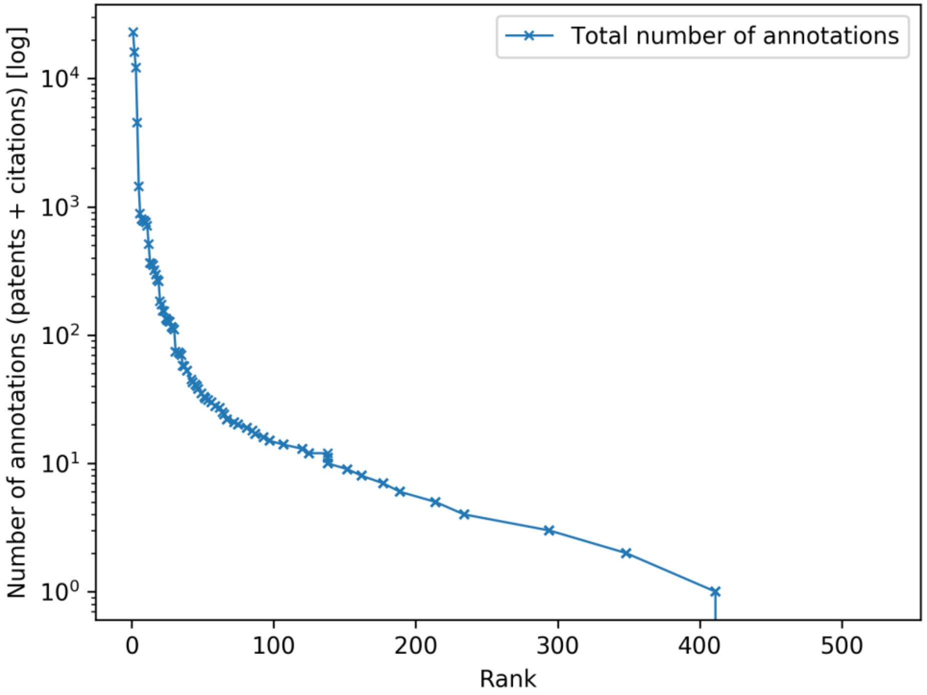

Supplementary Figure 1. Distribution of the total number of annotations (patents + citations). Compounds were ranked from highest number of annotations to lowest, and the number of annotations was plotted on a log scale. The corresponding data for this figure can be found in Supplementary Data 3.

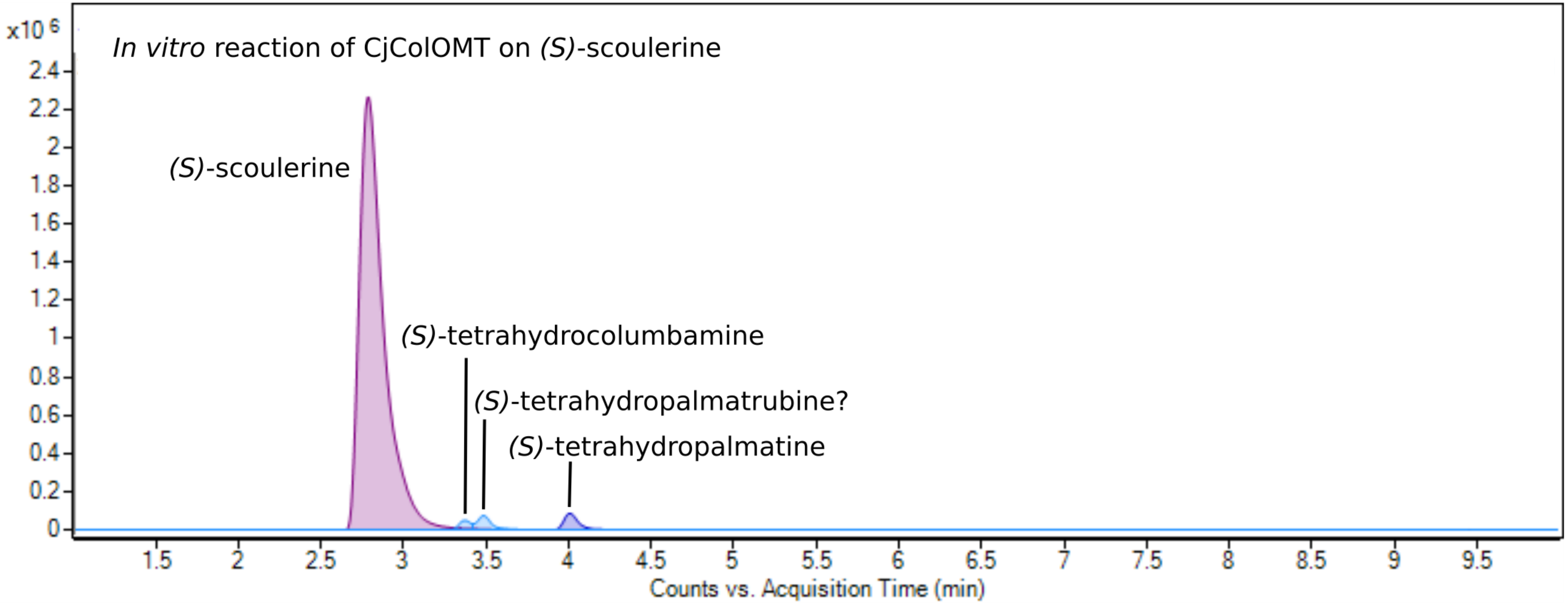

Supplementary Figure 2. LC-MS/MS traces of *in vitro* reaction of CjColOMT on (*S*)-scoulerine. The MRM transitions used for each compound are those described in Supplementary Table 7. The identities of (*S*)-scoulerine, (*S*)-tetrahydrocolumbamine, and (*S*)-tetrahydropalmatine were confirmed by coelution with authentic standards – an authentic standard for (*S*)-tetrahydropalmatrubine was not available, but its fragmentation pattern is consistent with that previously reported for (*S*)-tetrahydropalmatrubine[1].

Supplementary Reference

1. Valentic, T. R., Payne, J. T. & Smolke, C. D. Structure-guided engineering of a scoulerine 9-*O*-methyltransferase enables the biosynthesis of tetrahydropalmatrubine and tetrahydropalmatine in yeast. *ACS Catal.* **10, 8,** 4497–4509 (2020).
2. Galanie, S. & Smolke, C. D. Optimization of yeast-based production of medicinal protoberberine alkaloids. *Microb. Cell Fact.* **14**, 144 (2015).
3. Ryan, O. W. *et al.* Selection of chromosomal DNA libraries using a multiplex CRISPR system. *Elife* **3**, 1–15 (2014).
4. Srinivasan, P. & Smolke, C. D. Engineering a microbial biosynthesis platform for de novo production of tropane alkaloids. *Nat. Commun.* **10**, (2019).
5. Kotake, Y. *et al.* Detection and determination of reticuline and N-methylcoculaurine in the Annonaceae family using liquid chromatography-tandem mass spectrometry. *Journal of Chromatography B* **806**, 75–78 (2004).
6. Zou, S. *et al.* Simultaneous determination of five alkaloids by HPLC-MS/MS combined with micro-SPE in rat plasma and its application to pharmacokinetics after oral administration of lotus leaf extract. *Front. Pharmacol.* **10**, 1252 (2019).
7. Desgagné-Penix, I. & Facchini, P. J. Systematic silencing of benzylisoquinoline alkaloid biosynthetic genes reveals the major route to papaverine in opium poppy. *Plant J.* **72**, 331–344 (2012).
8. Díaz Chávez, M. L., Rolf, M., Gesell, A. & Kutchan, T. M. Characterization of two methylenedioxy bridge-forming cytochrome P450-dependent enzymes of alkaloid formation in the Mexican prickly poppy *Argemone mexicana*. *Arch. Biochem. Biophys.* **507**, 186–193 (2011).
9. Farrow, S. C., Hagel, J. M. & Facchini, P. J. Transcript and metabolite profiling in cell cultures of 18 plant species that produce benzylisoquinoline alkaloids. *Phytochemistry* **77**, 79–88 (2012).
