# Supplementary material for: A computational workflow for the expansion of heterologous biosynthetic pathways to natural product derivatives
Source: Nat Commun. 2021 Mar 19;12:1760. doi: 10.1038/s41467-021-22022-5 (PMC7979880; doi:10.1038/s41467-021-22022-5)
Supplement: Supplementary file 10 — Description of Additional Supplementary Files [file 41467_2021_22022_MOESM10_ESM.pdf]

Description of additional supplementary files

Title: Supplementary Data 1

Description: Compounds in biochemical network generated by BNICE.ch.

Title: Supplementary Data 2

Description: Reactions in biochemical network generated by BNICE.ch.

Title: Supplementary Data 3

Description: Popularity analysis for all BIA compounds in the network.

Title: Supplementary Data 4

Description: 50 most popular compounds in the generated network.

Title: Supplementary Data 5

Description: Sequences of codon-optimized genes used in this work.

Title: Supplementary Data 6

Description: Oligonucleotides used in this work.

Title: Supplementary Data 7

Description: Plasmids used in this work.
