# Supplementary material for: A computational workflow for the expansion of heterologous biosynthetic pathways to natural product derivatives
Source: Nat Commun. 2021 Mar 19;12:1760. doi: 10.1038/s41467-021-22022-5 (PMC7979880; doi:10.1038/s41467-021-22022-5)
Supplement: Supplementary file 11 — Reporting Summary [file 41467_2021_22022_MOESM11_ESM.pdf]

nature research

Corresponding author(s): Vassily Hatzimanikatis & Christina Smolke

Last updated by author(s): Feb 17, 2021

Reporting Summary

Nature Research wishes to improve the reproducibility of the work that we publish. This form provides structure for consistency and transparency in reporting. For further information on Nature Research policies, see our Editorial Policies and the Editorial Policy Checklist.

Statistics

For all statistical analyses, confirm that the following items are present in the figure legend, table legend, main text, or Methods section.

n/a	Confirmed	
☐	☒	The exact sample size (*n*) for each experimental group/condition, given as a discrete number and unit of measurement
☐	☒	A statement on whether measurements were taken from distinct samples or whether the same sample was measured repeatedly
☐	☒	The statistical test(s) used AND whether they are one- or two-sided<br>*Only common tests should be described solely by name; describe more complex techniques in the Methods section.*
☒	☐	A description of all covariates tested
☒	☐	A description of any assumptions or corrections, such as tests of normality and adjustment for multiple comparisons
☐	☒	A full description of the statistical parameters including central tendency (e.g. means) or other basic estimates (e.g. regression coefficient) AND variation (e.g. standard deviation) or associated estimates of uncertainty (e.g. confidence intervals)
☒	☐	For null hypothesis testing, the test statistic (e.g. F, t, r) with confidence intervals, effect sizes, degrees of freedom and P value noted<br>*Give P values as exact values whenever suitable.*
☒	☐	For Bayesian analysis, information on the choice of priors and Markov chain Monte Carlo settings
☒	☐	For hierarchical and complex designs, identification of the appropriate level for tests and full reporting of outcomes
☒	☐	Estimates of effect sizes (e.g. Cohen's *d*, Pearson's *r*), indicating how they were calculated

Our web collection on statistics for biologists contains articles on many of the points above.

Software and code

Policy information about availability of computer code

Data collection	Agilent MassHunter Workstation LC/MS Data Acquisition for 6400 Series Triple Quadrupole software (ver. B.08.02) was used for collection of LC-MS/MS data. Agilent MassHunter Workstation Optimizer for 6400 Series Triple Quadrupole software (ver. B.08.02) was used for development of multiple reaction monitoring parameters for chemical standards. NCBI BLAST online software (ver. 2.8.1) was used for alignment of DNA and amino acid sequences.
Data analysis	Agilent MassHunter Workstation Qualitative Analysis Navigator software (ver. B.08.00) and Quantitative Analysis software (ver. B.09.00) were used for analysis of all LC-MS/MS data. Microsoft Excel 2016 and Graphpad Prism 8 were used for analysis and statistical evaluation of all quantitative data and in the preparation of figures. Microsoft Word 2016 was used for preparation of the manuscript. Inkscape (ver. 0.92), Microsoft PowerPoint (ver. 16.16.20) and Adobe Illustrator (ver. 24.2.1) were used for final preparation of figures. MarvinVie) (ver. 6.2.2, ChemAxon) and PerkinElmer ChemDraw (ver. 19.1.1.32) were used for displaying chemical structures. Gephi (ver. 0.9.2) was used to visualize biochemical networks.

For manuscripts utilizing custom algorithms or software that are central to the research but not yet described in published literature, software must be made available to editors and reviewers. We strongly encourage code deposition in a community repository (e.g. GitHub). See the Nature Research guidelines for submitting code & software for further information.

Data

Policy information about availability of data

All manuscripts must include a data availability statement. This statement should provide the following information, where applicable:

- Accession codes, unique identifiers, or web links for publicly available datasets
- A list of figures that have associated raw data
- A description of any restrictions on data availability

Additional source data beyond those presented in this paper and the supplementary information files are available from the corresponding authors upon request.

Field-specific reporting

Please select the one below that is the best fit for your research. If you are not sure, read the appropriate sections before making your selection.

☒ Life sciences ☐ Behavioural & social sciences ☐ Ecological, evolutionary & environmental sciences

For a reference copy of the document with all sections, see nature.com/documents/nr-reporting-summary-flat.pdf

Life sciences study design

All studies must disclose on these points even when the disclosure is negative.

Sample size	No prior sample-size calculation was performed. All data represent measurements from three biological replicates, unless otherwise noted, where biological replicates represent independent microbial cultures grown from separate, individual colonies. As all engineering described in this work was performed at the cellular level and the metabolite titers collected are bulk measures of cellular populations, each independent microbial culture assayed thus represents a large population of individual cells. We therefore feel that three biological replicates are sufficient for the reliable measurement of altered metabolite production on the population level.
Data exclusions	One data point was excluded due to a failed injection on the LC-MS/MS, resulting in apparent values of 0 for all measured metabolites (including internal controls).
Replication	All measurements of metabolite titers presented in this work were repeated with multiple independent biological replicates (not technical replicates), each of which was inoculated from separate individual colonies, grown in separate containers, and independently assayed. All attempts at replication of results, both those shown in this work and those not shown, were successful.
Randomization	Randomization was not relevant to this study, as each biological sample assayed was a bulk population of microbial cells.
Blinding	Blinding was not relevant to this study, as each biological sample assayed was a bulk population of microbial cells.

Reporting for specific materials, systems and methods

We require information from authors about some types of materials, experimental systems and methods used in many studies. Here, indicate whether each material, system or method listed is relevant to your study. If you are not sure if a list item applies to your research, read the appropriate section before selecting a response.

Materials & experimental systems

n/a	Involved in the study	
☒	☐	Antibodies
☐	☒	Eukaryotic cell lines
☒	☐	Palaeontology and archaeology
☒	☐	Animals and other organisms
☒	☐	Human research participants
☒	☐	Clinical data
☒	☐	Dual use research of concern

Methods

n/a	Involved in the study	
☒	☐	ChIP-seq
☒	☐	Flow cytometry
☒	☐	MRI-based neuroimaging

Eukaryotic cell lines

Policy information about cell lines

Cell line source(s)	All cell lines described in this work are derived from wild-type Saccharomyces cerevisiae strain CEN.PK2-1D, which was obtained from EuroSCARF (30000B).
Authentication	The commercial cell line used in this study (see above) was not independently authenticated by the authors of this study.

Authentication	Authentication of any modifications made in engineered yeast strains was performed by DNA sequencing of the relevant genomic regions, as described in the Methods section of this study.
Mycoplasma contamination	Yeast cell lines were not tested for mycoplasma contamination.
Commonly misidentified lines<br>(See ICLAC register)	Not applicable.
